# The Emerging Role of Renal Tubular Epithelial Cells in the Immunological Pathophysiology of Lupus Nephritis

**DOI:** 10.3389/fimmu.2020.578952

**Published:** 2020-09-23

**Authors:** Seokchan Hong, Helen Healy, Andrew J. Kassianos

**Affiliations:** ^1^Division of Rheumatology, Department of Internal Medicine, Asan Medical Center, University of Ulsan College of Medicine, Seoul, South Korea; ^2^Conjoint Internal Medicine Laboratory, Chemical Pathology, Pathology Queensland, Health Support Queensland, Herston, QLD, Australia; ^3^Kidney Health Service, Royal Brisbane and Women's Hospital, Herston, QLD, Australia; ^4^Faculty of Medicine, University of Queensland, Brisbane, QLD, Australia

**Keywords:** lupus nephritis, renal tubular epithelial cells, tubulointerstitial lesions, kidney fibrosis, kidney inflammation

## Abstract

Systemic lupus erythematosus (SLE) is a systemic, autoimmune disease that can involve virtually any organ of the body. Lupus nephritis (LN), the clinical manifestation of this disease in the kidney, is one of the most common and severe outcomes of SLE. Although a key pathological hallmark of LN is glomerular inflammation and damage, tubulointerstitial lesions have been recognized as an important component in the pathology of LN. Renal tubular epithelial cells are resident cells in the tubulointerstitium that have been shown to play crucial roles in various acute and chronic kidney diseases. In this context, recent progress has been made in examining the functional role of tubular epithelial cells in LN pathogenesis. This review summarizes recent advances in our understanding of renal tubular epithelial cells in LN, the potential role of tubular epithelial cells as biomarkers in the diagnosis, prognosis, and treatment of LN, and the future therapeutic potential of targeting the tubulointerstitium for the treatment of patients with LN.

## Introduction

Systemic lupus erythematosus (SLE) is a chronic, systemic autoimmune disease that can affect and cause damage in various organs (1). The incidence and prevalence of SLE varies according to geographic and ethnic backgrounds, with the overall prevalence ranging from 3.2 to over 500 per 100,000 individuals (2). In terms of ethnic differences, the incidence and prevalence of SLE in African Americans, Hispanics, and Asians are ~2–5 times greater than in Caucasians (3). Further, females are predominantly affected during their childbearing years, with SLE identified as one of the leading causes of death in the young female population (4).

**Figure 1:**
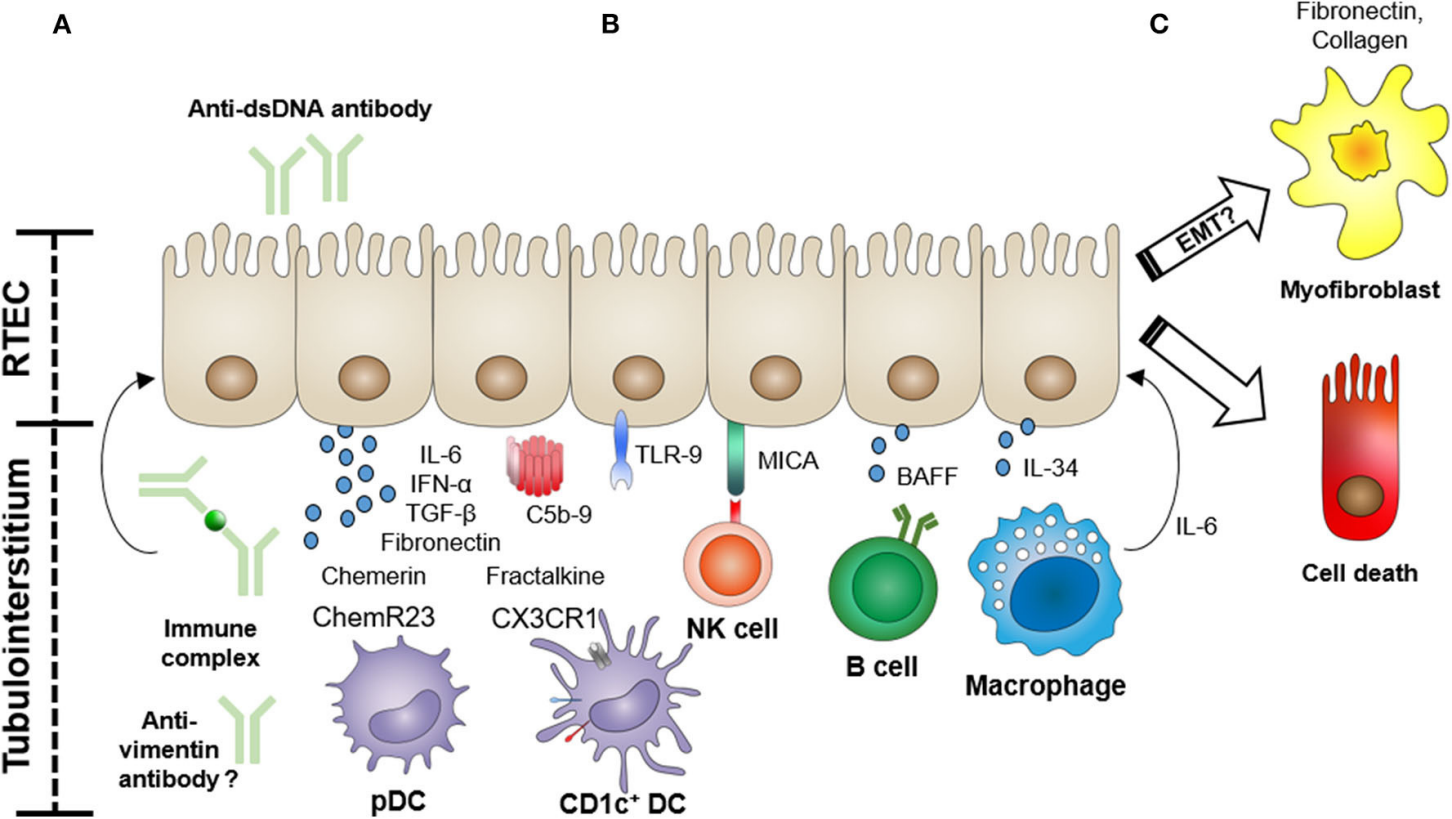
Functional role of renal tubular epithelial cells (RTECs) in the pathogenesis of lupus nephritis. RTECs actively participate in the tubulointerstitial pathology of lupus nephritis through the expression of cytokines, chemokines, and fibrogenic molecules, and *via* interactions with infiltrating immune cells. **(A)** RTECs can be activated by anti-dsDNA antibodies, immune complexes, or possibly anti-vimentin antibodies to produce pro-inflammatory cytokines and profibrotic molecules (e.g., fibronectin). Immune complex deposition in the tubular basement membrane can also lead to complement activation. **(B)** RTECs recruit and activate immune cells (e.g., dendritic cells (DCs), natural killer (NK) cells, B cells, and macrophages) *via* the production of chemotactic factors (e.g., chemerin and fractalkine) and expression of cell surface-activating molecules [MHC class I polypeptide-related sequence A (MICA)] and secreted cytokines [e.g., B-cell-activating factor (BAFF) and interleukin (IL)-34]. **(C)** In particular, recruited macrophages can initiate RTEC apoptosis *via* an IL-6-mediated mechanism, further driving tubular damage. The role of RTEC-mediated fibrogenesis *via* a process of epithelial–mesenchymal transition (EMT) is also under active investigation.

Lupus nephritis (LN), the involvement of SLE in the kidney, is one of the most common and severe manifestations of this autoimmune disease. The current International Society of Nephrology/Renal Pathology Society (ISN/RPS) pathological classification of LN is exclusively based on glomerular lesions (5). Tubulointerstitial inflammation, however, is frequently observed in LN, and several recent studies show that tubulointerstitial damage is a potent predictor for poor long-term renal outcomes in LN (6–9). Renal tubular epithelial cells (RTECs) are actively involved in the immune response in the kidney through the production of pro-inflammatory cytokines/chemokines and *via* interactions with immune cells (10). In this context, several studies have begun to examine the functional role of tubular epithelial cells in this autoimmune disorder. Here, we review the current knowledge on the role of RTECs in the pathogenesis of LN (Figure 1), relating the functional evidence provided from studies of experimental animal models to observations made in humans.

## Incidence and Prevalence of LN

LN occurs in up to 50% of SLE cases and is associated with increased morbidity and mortality compared with non-LN SLE patients. Although advances in diagnosis and treatment have been made, LN remains a significant cause of end-stage renal disease (ESRD), with more than 20% of patients with LN progressing to ESRD within 15 years of initial diagnosis (11). Indeed, the rates of developing ESRD have not improved and even tended to increase in recent decades. Given that the most common demographic affected by SLE is women of childbearing age, this has significant deleterious health and socioeconomic impacts (2). Further compounding this issue, clinical trials for therapeutics targeting LN have shown disappointing results thus far (12). This is, in part, due to our limited understanding of the cellular and molecular pathways driving the pathogenesis of LN.

## Initiation and Pathogenesis of LN

Intracellular material [e.g., chromatin, double-stranded DNA (dsDNA)] released during cell death plays a central role in the pathogenesis of SLE. The defective clearance of this cellular debris and loss of self-tolerance drives the production of antinuclear antibodies (e.g., anti-dsDNA antibodies) and formation of immune complexes (ICs) of self-nuclear antigens and its autoantibodies. Glomerular deposition of these ICs is considered the initiating step in the development of LN (13). In turn, this triggers a pro-inflammatory response characterized by complement activation and immune cell infiltration that drives the glomerular pathology of LN. However, a significant proportion (up to 97.6%) of SLE patients without overt proteinuria and/or renal dysfunction have glomerular lesions associated with histopathological deposition of ICs in the mesangium (14–16). Interestingly, these patients do not develop significant impairment in kidney function during long-term follow-up periods (15). These studies indicate that glomerular IC deposition alone is insufficient for the development of clinically significant LN, leading to recent research interest to examine the tubulointerstitial compartment in the pathogenesis of this disease.

## Tubulointerstitial Damage in LN

Although current histopathological classifications of LN are exclusively determined by the features and extent of glomerular lesions (5), other components of the kidney are also participants in the disease process (17). Tubulointerstitial damage is identified as one of the pathological features of the lupus kidney. Tubulointerstitial lesions are often associated with more severe (proliferative and sclerosing) forms of glomerular injury in LN (18, 19), with tubular atrophy and interstitial inflammation/fibrosis incorporated in assessments of active and chronic changes in LN (5). Recent research has also revealed the importance of tubulointerstitial damage in the prognosis of LN (6–9). In particular, tubulointerstitial inflammation and scarring are shown to be a more accurate predictor of long-term renal outcomes in LN than the glomerular-based ISN/RPS classification (7, 8). In a multivariate analysis of 105 patients with LN, tubulointerstitial lesions were significantly associated with the development of ESRD [hazard ratio (HR) 3.89, 95% confidence interval (CI) 1.25–12.14; *p* = 0.02], whereas glomerular histology based on ISN/RPS classes was not (*p* = 0.72) (8). Thus, understanding the mechanisms underlying tubulointerstitial inflammation is of importance in the study and treatment of LN.

In human LN, inflammatory cells have been shown to infiltrate the tubulointerstitium and form T:B cell aggregates or germinal center-like structures containing follicular dendritic cells (DCs) (20). In this study, Chang et al. analyzed the *in situ* immunoglobulin (Ig) repertoire, concluding that intrarenal B-cell clonal expansion was occurring within these tubulointerstitial germinal centers (20). Similarly, in lupus mice with nephritis, plasma cells secreting anti-dsDNA antibodies were primarily found in the kidney tubulointerstitium rather than the spleen or bone marrow (21). These collective findings identify the tubulointerstitial compartment as an important site of autoreactive B-cell immunity in LN.

IC deposits are identified along the tubular basement membrane (TBM) in LN patients. Wang et al. associated the presence of TBM deposits with more active disease, including higher serum creatinine levels and poorer prognosis in non-proliferative LN (22). Indeed, IC deposition in the TBM can lead to activation of the complement system, which, in turn, is associated with more severe tubulointerstitial pathology (interstitial fibrosis and tubular atrophy) (23). Interestingly, in a study of LN biopsies, the antibody subclass composition of TBM deposits was shown to differ from vascular and glomerular deposits, suggesting that tubular ICs are formed independently of ICs from the circulation and glomeruli (24). Examining the cellular and molecular pathways triggered by these tubular deposits and their utility as prognostic biomarkers is an emerging field of research focus.

## Renal Tubular Epithelial Cells in LN

RTECs are a kidney-resident population that responds and contributes to pathological processes in acute and chronic kidney diseases. RTECs can actively modulate tubulointerstitial immune cell responses (e.g., DCs, T cells, and B cells) through the production of soluble factors (e.g., pro-inflammatory cytokines and chemokines) and expression of cell-surface costimulatory and coinhibitory molecules (25–30). RTECs are, therefore, studied as a plausible dominant target of anti-dsDNA antibodies in the pathogenesis of LN.

## Anti-dsDNA Antibody and IC Triggering of RTECs

Early studies examining anti-dsDNA antibody triggering of RTECs used monoclonal antibodies (mAbs) derived from nephritic mice or SLE patients with clinically active LN. These seminal investigations showed that mouse and human antibodies to dsDNA, which also cross-react with small nuclear ribonucleoproteins (SnRNPs) A and D polypeptides, cause direct RTEC injury (31, 32). Interestingly, mouse mAb BWds3 was shown to bind to the cell surface of porcine RTEC line (PK-15) without penetration into the intracellular space, resulting in significant cell lysis. In contrast, mouse mAb BWdsl, which was internalized into the cytoplasm and nuclei of RTECs, displayed only a modest lytic effect (31). These findings suggest that anti-dsDNA antibodies that bind to the surface of RTECs, but without cellular penetration and cytoplasmic/nuclear translocation, have more pathogenic potential. A subsequent mechanistic study using mutants of mouse anti-dsDNA mAb 3E10 showed that penetration required only the F(ab) (antigen-binding fragment) portion, demonstrating a process of antibody internalization independent of Fc receptor-mediated binding (32).

The pathogenic interactions of anti-dsDNA antibodies with RTECs have also been investigated using polyclonal antibodies (pAbs) from sera of patients with LN. Compared with control IgG or non-anti-DNA IgG, binding of anti-dsDNA pAb from active LN patients to human RTECs induced secretion of pro-inflammatory cytokines [e.g., interleukin (IL)-6] (33). Another study by Yung et al. investigated the contribution of anti-dsDNA antibodies on fibrogenesis in RTECs (34). The excessive accumulation of extracellular matrix (ECM) proteins (e.g., collagen and fibronectin) is considered a histopathological hallmark of tubulointerstitial fibrosis (35). In their study, Yung et al. showed fibronectin to be highly expressed in the TBM of LN renal biopsies and colocalizing with antibody deposition. The group subsequently reported that anti-dsDNA pAb from LN patients triggered a significant increase in soluble and cell-associated fibronectin expression in human RTECs—a process dependent, in part, on the secretion of the profibrotic molecule transforming growth factor (TGF)-β by RTECs (34). This data suggests that fibrosis development in LN is initiated and amplified *via* complex signaling pathways involving anti-dsDNA antibodies, fibronectin, and TGF-β in RTECs.

It must also be noted that a characterization of *in situ*-expressed immunoglobulins from LN biopsy specimens identified vimentin, but not dsDNA, as the dominant target of humoral immunity in human lupus tubulointerstitial nephritis (36). In addition, previous studies have reported that titers of anti-dsDNA antibodies are not significantly associated with the degree of tubulointerstitial damage in patients with LN (7, 19). Thus, future studies addressing the effects of alternate autoantibodies (other than anti-dsDNA antibodies) on RTECs is required to better comprehend the *in vivo* tubulointerstitial pathophysiology of human LN.

## TLR Expression in RTECs

Emerging evidence reports that Toll-like receptors (TLRs) are actively involved in the pathogenesis of SLE and LN. In particular, nucleic acid-sensing TLRs such as TLR-3 (recognizing double-stranded RNA), TLR-7 (recognizing single-stranded RNA), and TLR-9 (recognizing dsDNA) have been implicated in the dysregulated immunity of LN, either responding to self-nucleic acids alone or in ICs (37). Initially described in innate immune cells (e.g., macrophages and DCs), TLRs are also expressed in non-immune cells. Expression of TLR-9 has been detected in the tubulointerstitium of patients with LN (38). Furthermore, significant correlations between the levels of TLR-9 expression in RTECs and tubulointerstitial damage have been reported in NZB/NZW lupus mice and LN patients (39). In this study, sera or ICs from SLE patients were shown to significantly induce TLR-9 in human RTECs (HK-2 cells) compared with those from healthy controls or undifferentiated connective tissue disease patients, although the difference in sera between SLE patients with and without LN was not addressed (39). This increased RTEC expression of TLR-9 was inhibited with short synthetic oligodeoxynucleotides, supporting an important stimulatory role for the DNA component within ICs in LN.

The role of other TLR classes in the tubulointerstitial pathology of LN is unclear and often complicated by findings in other renal diseases. For instance, TLR-4 signaling inhibits tubular damage, but also promotes fibrosis in a model of obstructive nephropathy (40). Given the importance of other TLRs (TLR-2 and TLR-4) in the development of glomerular injury in LN (41–43), functional evaluations of these TLRs in mediating the tubulointerstitial pathology of LN is now required.

## RTEC Cytokine Production in LN

Cytokines and chemokines contribute to LN immunopathogenesis, with the active role of RTECs as a source of these soluble factors of particular interest. Type I interferons (IFN-α/β) are considered to be pivotal in the pathogenesis of LN, with diverse effects on innate and adaptive immune cells (44). While plasmacytoid DCs (pDCs) are the major producer of type I IFN, expression in renal parenchymal cells has also been reported. In human biopsies with severe LN, RTECs have been identified as a key producer of IFN-α (45). In addition, a type I IFN-regulated signature was detected in RTECs, but not in the glomeruli, indicating a potential autocrine effect (45). Subsequent *in vitro* stimulation of RTECs with IFN-α was shown to induce expression of low-molecular mass protein-7 (LMP-7), a proteolytic subunit of the immunoproteosome that shapes the repertoire of antigenic peptides presented on major histocompatibility complex (MHC) class I molecules. Indeed, LMP7 was also highly expressed in renal tubules within biopsies from patients with severe LN (45). Interestingly, immunoproteasome inhibitors are emerging as promising therapeutic agents in the treatment of lupus (46).

B-cell-activating factor (BAFF) is another key cytokine in LN, essential for B-cell survival and maturation (47). RTECs have recently been identified as an important source of BAFF, with tubular expression in lupus-prone MRL-Fas^lpr^ mice and biopsies of patients with LN correlating with disease activity (48). In this study, *in vitro* functional assays with human RTECs revealed an autoamplification loop in which ligation of BAFF with its binding receptor (BAFF-R) induced colony-stimulating factor (CSF)-1 that, in turn, triggered further BAFF expression. In addition, BAFF stimulation was shown to augment cellular cytotoxicity in CSF-1-primed RTECs (48). These complex BAFF-dependent signaling pathways in RTECs may thus contribute to the established cell death and tubular atrophy observed in LN (49). Interestingly, belimumab, a mAb to BAFF, has shown promising results in the treatment of LN, although patients with severe LN were excluded from clinical trials (50).

The diagnostic and therapeutic potential of macrophage growth factor IL-34 in LN has also been examined. Serum IL-34 levels have been shown to correlate with SLE Disease Activity Index (SLEDAI) scores and distinguish between different histological classes of LN in patients with insignificant proteinuria, indicating its utility as a surrogate biomarker for subclinical LN (51). Wada et al. recently demonstrated robust RTEC expression of IL-34 in biopsies from LN patients and MRL-Fas^lpr^ lupus mice, with significant associations between expression levels and disease activity. Further mechanistic investigations using this lupus mouse model showed that IL-34 enhances intrarenal macrophage accumulation/proliferation, leading to macrophage-mediated RTEC apoptosis (52). These findings identify IL-34 as a novel therapeutic target of RTEC-mediated immunopathogenesis in LN.

## RTEC–Immune Cell Interactions in LN

Chemotactic and activatory signals between RTECs and tubulointerstitial immune cells are promising therapeutic targets in LN. RTEC recruitment of pDCs into the renal tubulointerstitium in human LN has been proposed *via* a chemerin–ChemR23 axis (53). In this study, De Palma et al. demonstrated human RTEC production of functionally active chemerin in response to pro-inflammatory cytokine tumor necrosis factor (TNF)-α, resulting in efficient recruitment of ChemR23^+^ pDC in transendothelial migration assays (53).

A dysregulated natural killer (NK) cell profile has been associated with the development of SLE. Reduced peripheral NK cell numbers and impaired cytotoxic functions have been reported in SLE patients, with NK cell deficiencies particularly prominent in patients with LN (54). However, circulating NK cells from patients with active SLE also have an activated phenotype, producing large amounts of pro-inflammatory cytokine IFN-γ (55). Evidence in LN patients of strong RTEC expression of MHC class I polypeptide-related sequence A (MICA), the activating ligand for NK receptor NKG2D, provides a possible mechanistic pathway for this human NK cell activation (56). In SLE-prone (MRL/MpJ and MRL/lpr) mice undergoing a lupus nephritic process, kidney NK cells similarly have an activated phenotype as demonstrated by IFN-γ production and signal transducer and activator of transcription 5 (STAT5) phosphorylation (56). STAT5 is a member of the STAT family of proteins, which signal *via* the Janus kinase (JAK)/STAT pathway, supporting the proposed application of JAK inhibitors for the treatment of LN (57).

Myeloid cells (e.g., DCs, monocytes/macrophages) also contribute to LN pathology via their specialized phagocytic and antigen-presenting functions, leading to inflammation and tubulointerstitial fibrosis (58). We have reported that TGF-β-expressing human CD1c^+^ myeloid DCs are recruited and retained in the renal tubulointerstitium *via* RTEC-derived fractalkine, providing evidence of a profibrotic RTEC–DC interaction (30). We have also recently proposed a pathogenic role for CD11c^+^ macrophages in the tubulointerstitial damage of LN (59). In the urine of LN patients, we identified a population of CD11c^+^ macrophages with an activated and pro-inflammatory phenotype, as defined by expression of costimulatory molecules (CD80, ICOSL, and OX40L) and cytokines (IL-6, IL-1β) (59). Furthermore, in this study, peripheral monocytes treated with sera from SLE patients acquired the identical phenotypic characteristics of these urinary CD11c^+^ macrophages and were shown in functional experiments to trigger IL-6-mediated fibronectin expression and apoptosis in human RTECs (59). This investigation supports the concept of a pathogenic role for RTEC–myeloid cell interactions in LN fibrogenesis.

## RTECs in Ln Fibrogenesis

As stated in preceding sections, RTECs can secrete profibrotic molecules (e.g., collagen, fibronectin) in response to anti-dsDNA antibody and inflammatory immune cells. A comprehensive study applying an unbiased single-cell RNA sequencing approach to kidney tissue from LN patients associated a fibrotic gene signature in tubular cells (upregulation of genes encoding ECM-related proteins—COL1A1, COL1A2, COL14A1, and COL5A2) with failure to respond to treatment (60). Of note, the fibrotic gene signature was detectable in a proportion of biopsies without tubulointerstitial fibrosis (as measured by standard histopathological assessment), suggesting that this identified signature may be of diagnostic utility for predicting tubulointerstitial damage prior to the development of overt fibrosis. Follow-up longitudinal studies with repeat biopsies are required to establish whether the presence of this fibrotic gene signature in tubular cells can indeed predict the development of renal fibrosis.

Tubular ECM expression has also been linked to the process of renal epithelial–mesenchymal transition (EMT), a mechanism of fibrogenesis during which RTECs differentiate into myofibroblasts and secrete ECM proteins (61, 62). While there is some controversy regarding tubular EMT, Liu et al. recently reported that oncostatin M, a member of the IL-6 cytokine family, could induce tubular EMT and fibrotic lesions in a murine model of LN (63). Further evaluation of this mechanism and tubular EMT in human LN is required.

Hypoxia has been proposed as one of the pathological drivers of injury in LN (64), with tubulointerstitial expression levels of hypoxia-inducible factor (HIF)-1α correlating with histopathological activity in patients with LN (65). Given that HIF-1α expression in RTECs can promote renal tubulointerstitial fibrosis (66), assessing the role of hypoxic RTECs in LN fibrogenesis will also be an important research area for future investigation.

## Therapeutic Approaches Targeting RTECs in LN

Given the pathogenic functions ascribed to RTECs in LN, therapeutic targeting of this tubular cell population (and its overexpressed molecules) has been an area of intense preclinical investigation. Fractalkine is one such molecule proposed for clinical targeting in LN. In addition to a previously highlighted function in kidney DC recruitment (30), Fu et al. provided evidence of a fractalkine–Wnt/β-catenin axis that promotes EMT progression and tubulointerstitial fibrosis in the kidneys of MRL/lpr mice and human RTECs (HK-2 cells) (67). Interestingly, the profibrotic Wnt/β-catenin signaling pathway is also increased in the tubular compartment of lupus-prone mice (proteinuric NZB/NZW mice), accompanied by elevated serum and renal levels of proapoptotic factor dickkopf-1 (Dkk-1) (68). It is proposed that the proapoptotic effects of Dkk-1 may perpetuate autoimmunity *via* release of chromatin-containing ICs (68). Thus, the development of novel treatments or repurposing of approved drugs targeting fractalkine [e.g., E6011, a humanized antifractalkine monoclonal antibody assessed in clinical trials for rheumatoid arthritis (69)] may be of therapeutic benefit in LN.

Kallikreins are a subgroup of serine proteases that exert multiple biological functions under normal and pathological conditions (e.g., hypertension, cancer, and inflammation) (70). Of particular relevance to LN, kallikreins and their end product, bradykinin, suppress type I IFN responses (71). Tissue kallikrein-1 (KLK1) is expressed in human RTECs under *in vitro* diseased conditions (72). The inducible expression of KLK1 in RTECs has also been shown to downmodulate local pathological reactions and confer renoprotection in mice with spontaneous LN (73). Further preclinical studies of KLK1 may support the evaluation of DM199, a recombinant form of human KLK1 currently in acute ischemic stroke clinical trials (74), for the treatment of LN.

## Conclusion

The collective findings from experimental mouse models and human clinical studies highlight the importance of tubulointerstitial damage in LN. In particular, RTECs are central effector cells within this local microenvironment, mediating renal pathology *via* the expression of pro-inflammatory/profibrotic molecules and through complex interactions with tubulointerstitial immune cells. This accumulating evidence provides important insights in understanding the mechanisms of RTEC-mediated pathology in LN, a potent predictor of longer-term renal outcomes. The broader application of these findings introduces novel approaches with the potential for greater prognostic and therapeutic specificity in targeting the tubulointerstitial expression of immunostimulatory molecules and EMT progression in human LN.

## Author Contributions

SH, HH, and AK drafted, revised, and approved the final version of the manuscript. All authors have participated sufficiently in the work to take public responsibility for the content.

## Conflict of Interest

The authors declare that the research was conducted in the absence of any commercial or financial relationships that could be construed as a potential conflict of interest.
